# Fungal endophytes associated with Inca nut plants (*Plukenetia volubilis* L.) in China and Thailand and their plant growth promoting properties

**DOI:** 10.3389/fmicb.2026.1794613

**Published:** 2026-05-19

**Authors:** Sheng Xu, Rungtiwa Phookamsak, Jianchu Xu, Jaturong Kumla, Nakarin Suwannarach

**Affiliations:** 1 Master of Science Program in Applied Microbiology (International Program), Faculty of Science, Chiang Mai University, Chiang Mai, Thailand; 2 Department of Biology, Faculty of Science, Chiang Mai University, Chiang Mai, Thailand; 3 Center of Excellence in Microbial Diversity and Sustainable Utilization, Chiang Mai University, Chiang Mai, Thailand; 4 Department of Economic Plants and Biotechnology, Yunnan Key Laboratory for Wild Plant Resources, Kunming Institute of Botany, Chinese Academy of Sciences, Kunming, China; 5 Honghe Center for Mountain Futures, Kunming Institute of Botany, Chinese Academy of Sciences, Honghe County, China; 6 Centre for Mountain Futures (CMF), Kunming Institute of Botany, Kunming, Yunnan, China; 7 CIFOR-ICRAF China Program, World Agroforestry (ICRAF), Kunming, China; 8 Office of Research Administration, Chiang Mai University, Chiang Mai, Thailand

**Keywords:** crop plant, endophytic fungi, Inca nut plant, phytohormones, plant growth promoting ability

## Abstract

Endophytic fungi have attracted increasing attention because of their roles in plant growth promotion and their potential applications in agriculture. This study aimed to isolate, identify, and evaluate the plant growth-promoting potential of endophytic fungi from healthy leaves of the Inca nut plants (*Plukenetia volubilis*) collected from three sites located in China (Honghe, Yunnan Province) and Thailand (Fang and Chiang Dao Districts, Chiang Mai Province). A total of 168 fungal strains belonging to 35 previously known genera and one unrecognized genus were obtained, of which Ascomycota were predominant, accounting for 166 strains (98.8%). The results showed that the Honghe site yielded the highest number of fungal strains. For fungal identification, the internal transcribed spacer (ITS) region was used as the molecular marker. *Nigrospora* and *Cercospora* predominated in the Honghe and Chiang Dao sites, respectively, whereas *Hypoxylon* was the dominant genus in the Fang site. Two genera, *Colletotrichum* and *Pseudopithomyces*, were found at all three sampling sites. A total of 96 strains tested positive for siderophore production, and 47 strains solubilized insoluble phosphate. Additionally, 13 fungal strains produced indole-3-acetic acid (IAA), among which *Colletotrichum* strain 1-8-1 yielded the highest concentration at 470.00 μg mL^−1^. The results showed that 57 strains tested positive for cellulase production and 10 strains for chitinase production. Moreover, fungal culture filtrates containing IAA from *Colletotrichum* strains 1-1-6, 1-8-1, and 2-1-15 enhanced seed germination in Chinese kale and tomato. Regarding seedling growth, the fungal culture filtrate of strain 1-8-1 also significantly increased root length and shoot height of Chinese kale and tomato seedlings. This is the first comprehensive study documenting endophytic fungi from Inca nut plants in China and Thailand. Furthermore, this study provides valuable insights into beneficial endophytic fungi associated with the Inca nut plant and highlights their potential applications in plant growth promotion.

## 1 Introduction

Endophytic fungi represent a remarkably varied and numerous groups of microorganisms that live symbiotically within host plants while causing no apparent harm or disease (Alam et al., 2021; Li et al., 2025; Liao et al., 2025). Initially discovered in the early 1800s, these fungi became the subject of considerable scientific interest during the 1990s due to their protective functions, helping host plants defend against disease-causing organisms, harmful insects, and pathogens (Caruso et al., 2022; Fontana et al., 2021; Geng et al., 2022). Through their plant growth promoting characteristics, these fungi stimulate plant development via both direct and indirect pathways. Direct enhancement involves the uptake of nutrients from soil and the production of plant hormones, such as auxins (primarily indole-3-acetic acid; IAA), gibberellins, and cytokinins. Indirect promotion of growth is achieved through processes including the activation of systemic resistance mechanisms, the development of acquired systemic resistance, antibiotic generation, siderophore production, and alleviation of environmental stress (Baron and Rigobelo, 2022). For instance, endophytic *Trichoderma* species not only provide protection against pathogenic fungi and nematodes but also promote plant growth (Al-Ani et al., 2025; Rajini et al., 2020). *Phoma glomerata*, isolated from cucurbit plants, enhances rice growth through IAA-mediated mechanisms and improved nutrient assimilation (de Carvalho et al., 2020; Waqas et al., 2012). *Serendipita indica*, a root endophytic fungus, has been shown to improve drought tolerance and enhance the growth of walnut (*Juglans regia*; Zheng et al., 2024), as well as to promote the growth of rape (*Brassica napus*) by improving phosphorus acquisition through enhanced phosphate solubilization and uptake (Wu et al., 2018). The endophytic strain STL3G74 (*Aspergillus niger*) was found to produce IAA and siderophores, which together markedly enhanced the growth of English ryegrass (*Lolium perenne*; Tang W. et al., 2023). Colonization by the endophytic fungus *Fusarium avenaceum* in Kentucky bluegrass (*Poa pratensis*) has also been shown to improve mineral nutrient acquisition, leading to increased accumulation of Fe, Ni, and Zn (García-Latorre et al., 2023). *Aspergillus* sp. GMBUCC 24-013, a seaweed-associated endophytic fungus, has been shown to exhibit strong antagonistic activity against phytopathogenic fungi (*Neopestalotiopsis cubana* and *Colletotrichum siamense*), thereby indirectly promoting plant growth by reducing pathogen pressure and enhancing plant health (Abeygunawardane et al., 2025). However, research on fungal endophytes with the ability to promote plant growth continues to increase, driven by the escalating demand for agricultural productivity and the need to reduce or replace agrochemical use, thereby mitigating undesired consequences on the environment and human health (Poveda et al., 2021).

Inca nut (*Plukenetia volubilis*), commonly known as sacha inchi or Inca peanut, a perennial climbing oilseed crop of the family Euphorbiaceae, is indigenous to the Amazon rainforest of South America (Kodahl and Sørensen, 2021). This plant demonstrates robust environmental adaptability, thriving at temperatures between 10°C and 36°C and at altitudes ranging from 200 to 1,500 meters above sea level. It is a heliophilic plant and grows well in diverse soil types, with a relatively short cultivation cycle of 6–8 months to fruiting (Rodzi and Lee, 2022). Currently, it is cultivated in Peru, China, and Southeast Asian countries, with Peru remaining the global leader in production (Kodahl and Sørensen, 2021). The seeds of this plant have been used for cold-press oil extraction (Redjeki et al., 2025). Inca nut oil is widely utilized due to its high content of essential fatty acids, predominantly α- and γ-linolenic acids, which provide significant human health benefits (Le et al., 2024; Lupi et al., 2024; Srichamnong et al., 2018). In pharmaceutical applications, Inca nut oil has been traditionally used in dermatological care for skin emollience, wound healing, and the management of insect bites and cutaneous infections (Rodzi and Lee, 2022). It also exhibits various biological activities, including antimicrobial, anti-inflammatory, and anti-aging effects (Wang et al., 2018). Prior to this study, research on fungi associated with Inca nut had mostly focused on arbuscular mycorrhizal fungi (Tian et al., 2013; Wiriya et al., 2020) and the rhizosphere soil fungal community (Márquez-Dávila et al., 2020; Uwaremwe et al., 2024). Endophytic fungi in seven *Plukenetia* species, including Inca nut, have only been studied in Peru (Márquez-Dávila et al., 2020). However, research on endophytic fungi associated with this plant in China and Thailand remains limited. Therefore, this study aimed to isolate endophytic fungi from Inca nut plants cultivated in Yunnan, China and northern Thailand. The isolated fungi were identified using the ITS region as a molecular marker, and their taxonomic composition was analyzed. Their plant growth-promoting traits, including IAA and siderophore production, insoluble phosphate solubilization, and extracellular enzyme activities were evaluated. In addition, seed germination assays were conducted to assess the potential of selected endophytic fungal culture filtrates to promote early plant growth. These findings will contribute to the development of fungal stimulators and biofertilizers as sustainable alternatives to synthetic fertilizers, thereby promoting environmentally friendly and sustainable agricultural practices.

## 2 Materials and methods

### 2.1 Sample collection

**Figure 1:**
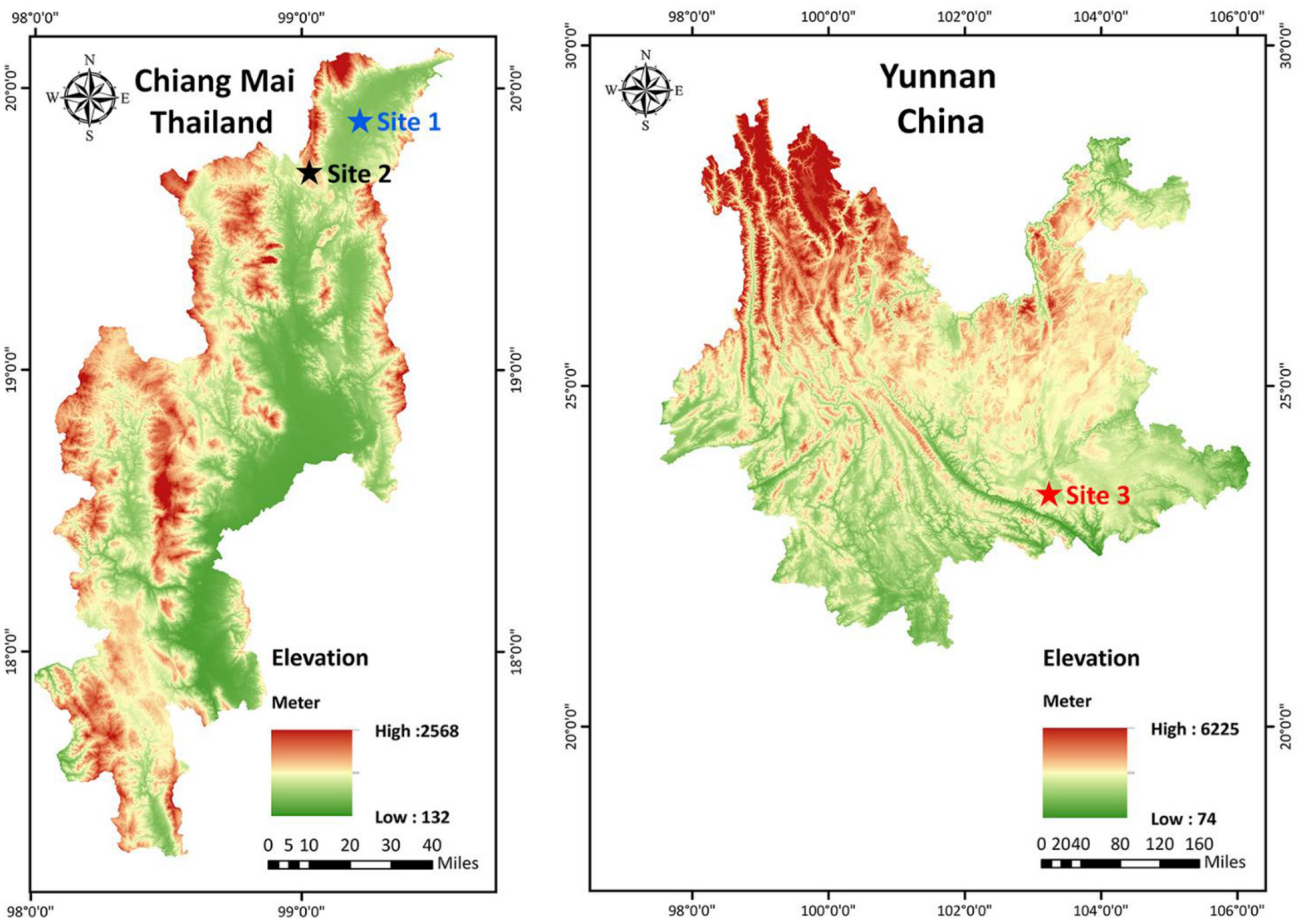
Geographical distribution of sampling sites for fresh, healthy leaves of Inca nut. Site 1: Chiang Dao District, Chiang Mai Province, Thailand; Site 2: Fang District, Chiang Mai Province, Thailand; Site 3: Honghe Prefecture, Yunnan Province, China.

Fresh, healthy leaves of Inca nut were collected from three locations. One site was in Honghe Prefecture, Yunnan Province, China (23°5′11.24″N, 103°4′15.56″E, elevation 550 m) during March 2021. Two sites were in Chiang Mai Province, Thailand, that is Chiang Dao District (19°3′13.01″N, 99°3′9.45″E, elevation 500 m) and Fang District (19°2′18.77″N, 991′45.61″E, elevation 1,000 m) during March 2022 (Figure 1). At each collection site, a stratified random sampling method was employed. 10 healthy plants were selected, and from each plant, 10 leaves were systematically collected from different canopy positions (upper, middle, and lower strata), resulting in a total of 100 leaves per site. Immediately after collection, the specimens were placed in sterile plastic bags and stored under ice boxes. Endophytic fungal isolation was initiated within 24 h of sampling.

### 2.2 Isolation of endophytic fungi

Endophytic fungi were isolated using the method described by (Suwannarach et al. 2012), with some modifications. Leaf specimens were thoroughly rinsed under running tap water and air-dried. Surface sterilization was performed in three sequential steps: immersion in 75% ethanol for 30 s, treatment with 5% sodium hypochlorite (NaClO) for 2 min, and a final immersion in 75% ethanol for 30 s. Subsequently, the samples were rinsed three times with sterile distilled water. Surface-sterilized leaves were aseptically cut into approximately 1 × 1 cm pieces using sterile scalpel. Six pieces from each surface-sterilized leaf were placed on potato dextrose agar (PDA) supplemented with 0.03% (*w*/*v*) rose bengal and 0.05% (*w*/*v*) chloramphenicol. Then, the isolation plates were incubated at 25°C under dark conditions for 5 days. Emerging hyphal tips from each sample were subcultured onto fresh PDA plates for purification. Pure cultures of endophytic fungi obtained through this process were preserved in 15% glycerol at −80°C for long-term storage at the Research Laboratory for Excellence in Sustainable Development of Biological Resources, Faculty of Science, Chiang Mai University (SDBR-CMU), Thailand. All fungal strains were used for further molecular identification and *in vitro* evaluation of plant growth-promoting traits.

### 2.3 Molecular identification of endophytic fungi

Fungal genomic DNA was extracted from the mycelia of each pure culture grown on PDA. After 7 days of cultivation, fungal mycelia were harvested using sterile scalpel. Genomic DNA was extracted from each fungal isolate using the Biospin Fungus Genomic DNA Extraction Kit (BioFlux, Hangzhou, China) following the manufacturer's protocol. The nuclear ribosomal internal transcribed spacer (ITS) region of rDNA was amplified as the molecular marker using the ITS5 and ITS4 primers (White et al., 1990). Polymerase chain reaction (PCR) was conducted in 25 μL reaction volumes containing: 8.5 μL of sterilized double distilled water (ddH_2_O), 12.5 μL of 2 × Power *Taq* PCR Master Mix (Easy*Taq*TM DNA Polymerase, dNTPs, and optimized buffer), 2 μL of DNA template, and 1 μL of each forward and reverse primer. Thermocycling parameters followed (Xu et al. 2022): initial denaturation at 94°C for 5 min; 35 cycles of 94°C for 30 s, 55°C for 45 s, and 72°C for 1 min; final extension at 72°C for 10 min. PCR products were visualized *via* 1% agarose gel electrophoresis and commercially sequenced by Tsingke Biotechnology Co., Ltd. (Beijing, China). Raw sequences were assembled and edited in SeqMan v3.1 (DNASTAR Lasergene) to generate consensus sequences. Taxonomic affiliations were determined through BLASTn queries against the NCBI GenBank database (access date: 10th September 2025).

### 2.4 Phylogenetic analyses

In the identification of endophytic fungi, the ITS locus is commonly employed as a marker for identification at the genus level (Dos Reis et al., 2022; Du et al., 2025). DNA sequences of Chytridiomycota (*Phlyctochytrium californicum* CBS 667.73 and *P. africanum* CBS 454.65) were used as outgroups following recent publications (Du et al., 2025). RAxML analysis was conducted based on the ITS region for 168 strains in this study. Sequence datasets were aligned *via* the online platform, MAFFT v. 7.526 (https://mafft.cbrc.jp/alignment/server/; Rozewicki et al., 2019). Ambiguous sites were trimmed using TrimAl v. 1.3 in the alignment tool on the web phylemon2 (http://phylemon.bioinfo.cipf.es/utilities.html, access on 20th September 2025). The aligned sequence matrix of the individual genes was prior analyzed by RAxML-HPC v.8 on ACCESS (8.2.12) tool *via* the web CIPRES Science Gateway v. 3.3 (Miller et al., 2010) for checking the congruent tree topologies. Parameters of maximum likelihood (ML) were set up as default settings but adjusted by selecting the GTR+GAMMA model of nucleotide substitution and 1,000 rapid bootstrap iterations. At the same time, the circular RAxML tree of 168 strains based on the ITS region was edited and visualized on Interactive Tree of Life (iTOL) v6 (https://itol.embl.de/, accessed on 21st September 2025; Letunic and Bork, 2024). All newly generated sequences from this study have been deposited in GenBank (https://www.ncbi.nlm.nih.gov/genbank, assessed on 12th January 2026).

### 2.5 Determination of plant growth promoting properties

#### 2.5.1 Siderophore production

Siderophore production was qualitatively assessed using the chrome azurol S (CAS) agar plate assay, following the method described by (Vellore 2001). A fungal plug from each strain, grown on PDA at 25°C for five days, was centrally inoculated onto CAS agar and incubated at 30°C in the dark for seven days. The formation of yellow, orange, or pink color-change zones surrounding fungal colonies was considered indicative of positive siderophore production. Uninoculated CAS agar plate served as negative control. Quantitative analysis of siderophore production was performed using the siderophore production index (SPI), calculated as the diameter of the color-change zone divided by the colony diameter. Three replicates were prepared for each fungal strain.

#### 2.5.2 Phosphate solubilization

Fungal plugs of each strain were inoculated onto Pikovskaya's (PVK) agar and incubated at 30°C for five days. The PVK agar was prepared according to (Mirsam et al. 2021) with the following composition per liter: 10 g glucose, 2.5 g Ca_3_(PO_4_)_2_, 0.5 g (NH_4_)_2_SO_4_, 0.2 g NaCl, 0.1 g MgSO_4_·7H_2_O, 0.2 g KCl, 0.002 g MnSO_4_·H_2_O, 0.002 g FeSO_4_·7H_2_O, 0.5 g yeast extract, and 15 g agar. Phosphate solubilization activity was evaluated by measuring the halo formation around the fungal colonies, as described by (Rosli et al. 2020). Uninoculated PKV agar plate served as negative control. The phosphate solubilization index (PSI) was calculated as the diameter of the halo zone divided by the colony diameter. Three replicates were prepared for each fungal strain.

#### 2.5.3 Indole compound production and indole-3-acetic acid (IAA) quantification

Each fungal strain was cultured in potato dextrose broth supplemented with 0.1% (w/v) L-tryptophan in Erlenmeyer flasks and incubated at 25°C on a shaker at 100 rpm, following the method described by (Patel et al. 2018). After five days of incubation, culture supernatants were collected by centrifugation at 8,000 × g for 15 min at 4°C. Then, 1 mL of culture supernatant was mixed with 2 mL of Salkowski reagent (Gordon and Weber, 1951), with the development of a pink-red color indicating indole compound production. Uninoculated liquid medium served as negative control. Subsequently, IAA in the fungal supernatant was quantified using high performance liquid chromatography (HPLC) following the protocol described by Kumla et al. (2020).

HPLC analysis was conducted using a Shimadzu Prominence Ultra-Fast Liquid Chromatography (UFLC) system (Shimadzu Corporation, Kyoto, Japan). Separation was achieved on a Mightysil RP-18 reversed-phase analytical column (250 mm × 4.6 mm, 5 μm particle size) maintained at 35°C. The mobile phase consisted of two components: (A) 2.5% (v/v) acetic acid in deionized water (pH 3.8 adjusted with KOH) and (B) 80% (v/v) acetonitrile in deionized water. The following gradient program was used: 0–22 min, 0–30% B; 23–25 min, increased to 50% B; 26–30 min, increased to 100% B. The flow rate was maintained at 0.5 mL min^−1^ with UV detection at 280 nm. The sample injection volume was 10 μL. IAA was identified by comparing retention times and spectra with the authenticated IAA standard. The fungal IAA was quantified with the calibration curve constructed with IAA standard. All the analyses were carried out in triplicate.

#### 2.5.4 Extracellular enzyme production

Production of cellulolytic and chitinolytic enzymes was evaluated using cellulose agar and colloidal chitin agar, respectively following the methods of previous studies (Shahriarinour et al., 2011; Subramanian et al., 2020). A fungal plug from each strain was inoculated at the center of each test plate and incubated at 30°C for 10 days. Then, the plates were stained with 1% (w/v) Congo red solution for 15 min, followed by destaining with 1 M NaCl solution. The formation of hydrolytic halos (translucent zones) around colonies indicated enzymatic activity. The enzyme activity index (EAI) was calculated following (Chaiya et al. 2021) as the diameter of the hydrolytic halo zone divided by the colony diameter. Three replicates were prepared for each fungal strain.

### 2.6 Plant growth promoting effects of selected fungal culture filtrates containing IAA

#### 2.6.1 Preparation of fungal culture filtrates

Each fungal supernatant was filtered through a 0.22 μm membrane filter and diluted with sterile distilled water to adjust the IAA concentration to 25 μg mL^−1^. An IAA solution at 25 μg mL^−1^ was used as a positive control, while sterile distilled water and PDB supplemented with 0.1% (w/v) L-tryptophan were used as negative controls.

#### 2.6.2 Seed germination

The three fungal culture filtrates with the highest IAA levels were selected and used in this experiment. Chinese kale (*Brassica oleracea* var. *alboglabra*) and tomato (*Solanum lycopersicum*) seeds were used. Seeds were surface disinfected by immersion in 1% NaClO for 1 min, followed by three rinses with sterile water. Then, 50 seeds of each plant were placed on sterile filter paper in 90-mm Petri dishes and supplemented with 5 mL of each solution. The plates were incubated in a growth chamber at 25°C with a 12-h light period. Germination of Chinese kale seeds was assessed after 24 h, whereas tomato seed germination was evaluated after 48 h, corresponding to complete germination for each plant. The germination index (GI) was determined following the method described by (Sadeghi et al. 2011). Each treatment was conducted in triplicate, with the experiment repeated twice under the same biological conditions.

#### 2.6.3 Seedling growth

The fungal culture filtrate that exhibited the highest seed germination rate was selected and used in this experiment. Both Chinese kale and tomato seedlings were surface disinfected as described above. Then, 30 seeds of each plant were placed on sterile filter paper in Petri dishes and supplemented with 5 mL of each solution. The plates were incubated in a growth chamber at 25°C with a 12-h light period. After five days of incubation, root length and shoot height of all seedlings were measured. Each treatment was conducted in triplicate, with the experiment repeated twice under the same biological conditions.

### 2.7 Statistical analysis

Data were analyzed by one-way analysis for variance (ANOVA) carried out with the SPSS program version 26.0 for Windows. The significant differences (*p* ≤ 0.05) between the mean value of each treatment were considered statistically significant using Duncan's multiple range test.

## 3 Results

### 3.1 Isolation, initial identification, and composition of endophytic fungi

**Figure 2:**
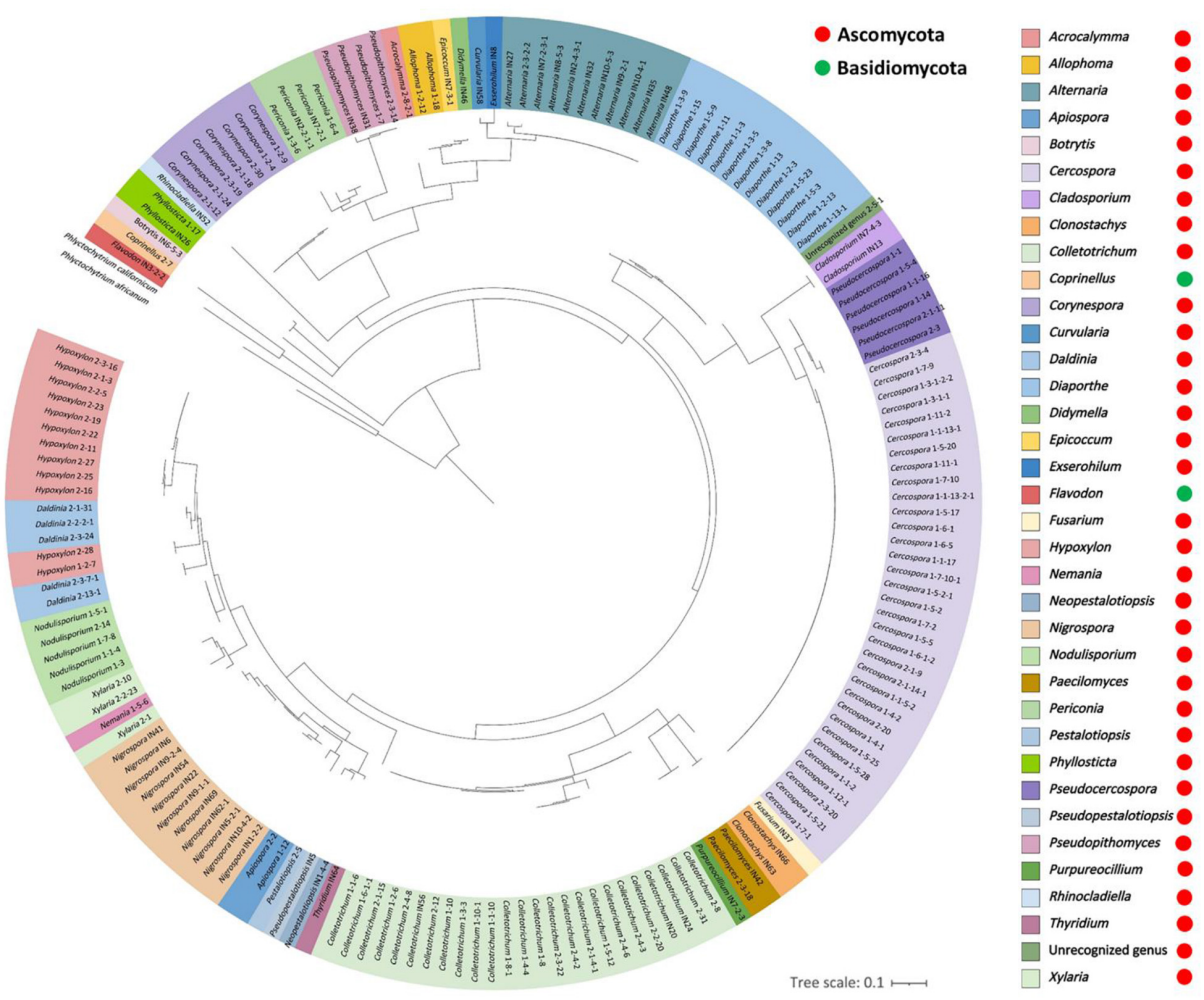
Circular phylogenetic tree showing the placement of fungal isolates at the generic level. The tree was generated using maximum likelihood based on 170 ITS sequences and visualized in ITOL. Different colors indicate independent genera, and the outgroups are *Phlyctochytrium californicum* (CBS 667.73) and *P. africanum* (CBS 454.65; Chytridiomycota).

**Figure 3:**
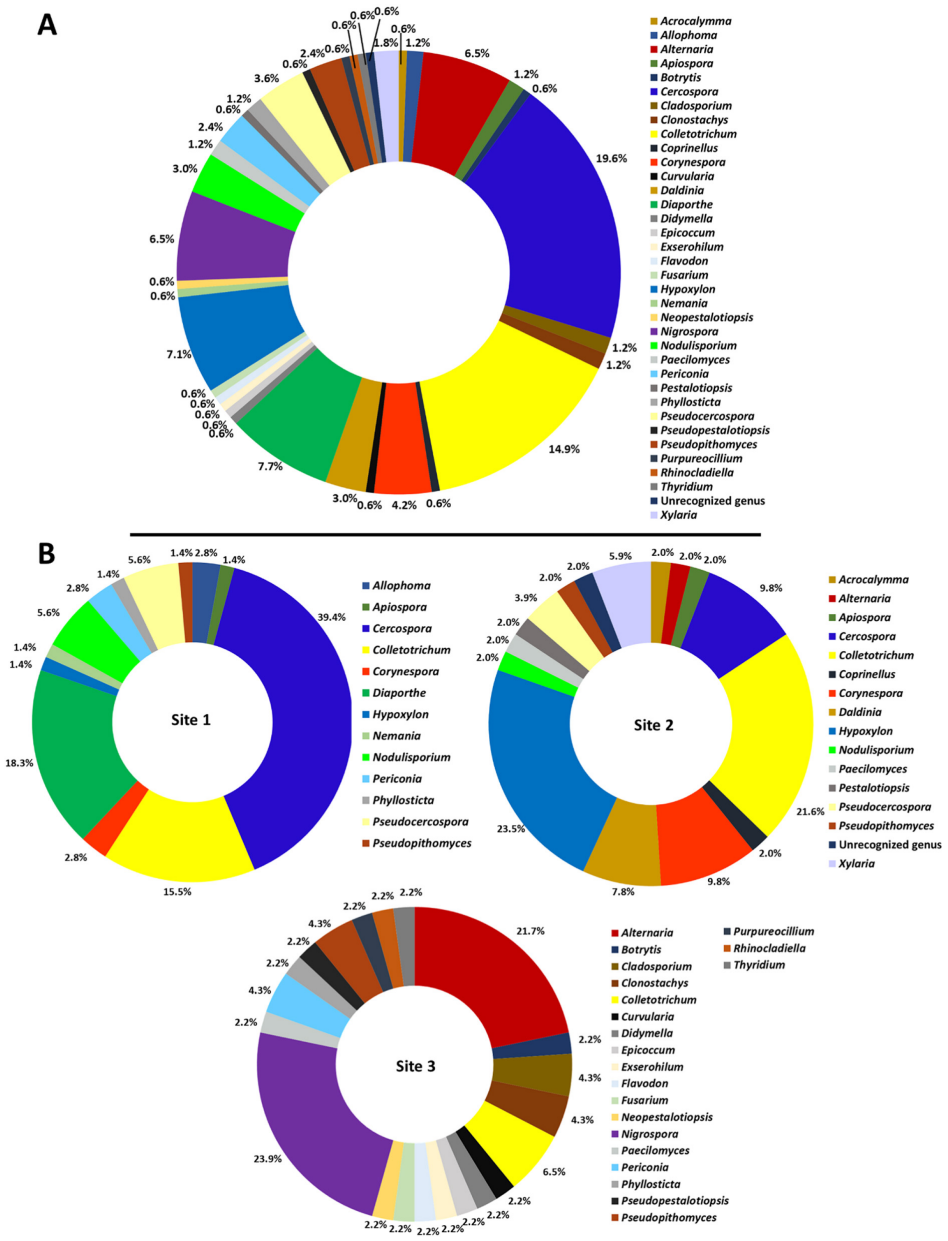
Generic composition of endophytic fungi isolated from Inca nut plants in this study. Overall generic composition across the three sampling sites **(A)** and individual sites **(B)**. Site 1: Chiang Dao District, Chiang Mai Province, Thailand; Site 2: Fang District, Chiang Mai Province, Thailand; Site 3: Honghe Prefecture, Yunnan Province, China.

A total of 168 endophytic fungal strains were obtained, of which 71, 51, and 46 strains were collected from site 1 (Chiang Dao District, Chiang Mai Province, Thailand), site 2 (Fang District, Chiang Mai Province, Thailand), and site 3 (Honghe Prefecture, Yunnan Province, China), respectively. Details on the fungal strains, their familial placement, and GenBank accession numbers are provided in Table 1. A total of 167 fungal strains were identified at the genus level based on ITS sequences and were classified into 35 previously known genera, belonging to 24 families across two phyla (Ascomycota and Basidiomycota). Additionally, the remaining strain, 2-5-1, was categorized as a previously unrecognized genus within Ascomycota based on its low sequence similarity (85.08%) to entries in the GenBank database; further assessments are required for its identification. A phylogenetic tree showing the genus-level placements based on ITS region of the fungal taxa is presented in Figure 2. The results indicated that Ascomycota is the dominant group in this study, with 166 strains from 34 genera of 22 families (Figure 2). The 33 genera identified within Ascomycota include *Acrocalymma, Allophoma, Alternaria, Apiospora, Botrytis, Cercospora, Cladosporium, Clonostachys, Colletotrichum, Corynespora, Curvularia, Daldinia, Diaporthe, Didymella, Epicoccum, Exserohilum, Fusarium, Hypoxylon, Nemania, Neopestalotiopsis, Nigrospora, Nodulisporium, Paecilomyces, Periconia, Pestalotiopsis, Phyllosticta, Pseudocercospora, Pseudopestalotiopsis, Pseudopithomyces, Purpureocillium, Rhinocladiella, Thyridium*, and *Xylaria*. Meanwhile, Basidiomycota accounted for two genera (*Coprinellus* and *Flavodon*). Of the total fungal isolates, *Cercospora* was the dominant genus (19.6%), followed by *Colletotrichum* (14.9%), *Diaporthe* (7.7%), *Hypoxylon* (7.1%), and *Nigrospora* (6.5%; Figure 3A).

**Table 1:** Site, strain, ITS GenBank number, closest species and generic name of endophytic fungi isolated from Inca nut plants in this study.

Site number	Stain SDBR-CMU	GenBank number	Closest species	Similarity (%)	Generic identification
Site 1	1-1	PX856364	*Pseudocercospora paracydoniae* CBS 149367	99.81	*Pseudocercospora*
	1-10	PX856421	*Colletotrichum siamense* MFLU 090230	100	*Colletotrichum*
	1-10-1	PX856422	*Colletotrichum siamense* ICMP:18578	100	*Colletotrichum*
	1-11	PX856423	*Diaporthe passifloricola* CPC 27480	99.11	*Diaporthe*
	1-1-10	PX856370	*Colletotrichum siamense* ICMP:18578	100	*Colletotrichum*
	1-11-1	PX856424	*Cercospora manoa* ICMP 21762	100	*Cercospora*
	1-11-2	PX856425	*Cercospora manoa* ICMP 21762	100	*Cercospora*
	1-1-13-1	PX856371	*Cercospora alyssopsidis* IRAN 3739C	100	*Cercospora*
	1-1-13-2-1	PX856372	*Cercospora musigena* CPC 24809	100	*Cercospora*
	1-1-16	PX856373	*Pseudocercospora assamensis* CBS 122467	100	*Pseudocercospora*
	1-1-17	PX856374	*Cercospora musigena* CPC 24809	100	*Cercospora*
	1-12	PX856426	*Apiospora cannae* ZHKUCC 22-0127	100	*Apiospora*
	1-1-2	PX856365	*Cercospora manoa* ICMP 21762	100	*Cercospora*
	1-12-1	PX856427	*Cercospora glycinicola* CPC 23912	100	*Cercospora*
	1-13	PX856428	*Diaporthe rosae* MFLUCC 17-2658	99.82	*Diaporthe*
	1-1-3	PX856366	*Diaporthe passifloricola* CPC 27480	99.10	*Diaporthe*
	1-13-1	PX856429	*Diaporthe rosae* MFLUCC 17-2658	100	*Diaporthe*
	1-14	PX856430	*Pseudocercospora assamensis* CBS 122467	99.61	*Pseudocercospora*
	1-1-4	PX856367	*Nodulisporium verrucosum* CBS:245.29	100	*Nodulisporium*
	1-15	PX856431	*Diaporthe yunnanensis* CGMCC 3.18289	100	*Diaporthe*
	1-1-5-2	PX856368	*Cercospora manoa* ICMP 21762	100	*Cercospora*
	1-1-6	PX856369	*Colletotrichum rhizophorae* MFLUCC 17-1927	99.82	*Colletotrichum*
	1-17	PX856432	*Phyllosticta rhizophorae* NCYUCC 19-0352	100	*Phyllosticta*
	1-18	PX856433	*Allophoma siamensis* MFLUCC 17-2422	100	*Allophoma*
	1-2-12	PX856380	*Allophoma minor* CBS:325.82	100	*Allophoma*
	1-2-13	PX856381	*Diaporthe rosae* MFLUCC 17-2658	99.82	*Diaporthe*
	1-2-3	PX856375	*Diaporthe rosae* MFLUCC 17-2658	99.82	*Diaporthe*
	1-2-4	PX856376	*Corynespora cassiicola* VIC:44233	100	*Corynespora*
	1-2-6	PX856377	*Colletotrichum aenigma* ICMP 18608	100	*Colletotrichum*
	1-2-7	PX856378	*Hypoxylon fendleri* BCC32408	99.83	*Hypoxylon*
	1-2-9	PX856379	*Corynespora cassiicola* VIC:44233	100	*Corynespora*
	1-3	PX856382	*Nodulisporium verrucosum* CBS:245.29	99.80	*Nodulisporium*
	1-3-1-1	PX856383	*Cercospora manoa* ICMP 21762	100	*Cercospora*
	1-3-1-2-2	PX856384	*Cercospora manoa* ICMP 21762	100	*Cercospora*
	1-3-3	PX856385	*Colletotrichum siamense* ICMP:18578	100	*Colletotrichum*
	1-3-5	PX856386	*Diaporthe chinensis* MFLUCC 19-0101	100	*Diaporthe*
	1-3-6	PX856387	*Periconia ananasi* MFLUCC 21-0155	99.64	*Periconia*
	1-3-8	PX856388	*Diaporthe chinensis* MFLUCC 19-0101	100	*Diaporthe*
	1-3-9	PX856389	*Diaporthe citri* LGMF946	98.77	*Diaporthe*
	1-4-1	PX856390	*Cercospora manoa* ICMP 21762	100	*Cercospora*
	1-4-2	PX856391	*Cercospora manoa* ICMP 21762	100	*Cercospora*
	1-4-4	PX856392	*Colletotrichum siamense* ICMP:18578	100	*Colletotrichum*
	1-5-1	PX856393	*Nodulisporium verrucosum* CBS:245.29	99.80	*Nodulisporium*
	1-5-12	PX856400	*Colletotrichum plurivorum* CBS 125474	99.81	*Colletotrichum*
	1-5-17	PX856401	*Cercospora musigena* CPC 24809	100	*Cercospora*
	1-5-2	PX856394	*Cercospora manoa* ICMP 21762	100	*Cercospora*
	1-5-20	PX856402	*Cercospora glycinicola* CPC 23912	100	*Cercospora*
	1-5-21	PX856403	*Cercospora manoa* ICMP 21762	100	*Cercospora*
	1-5-23	PX856395	*Diaporthe rosae* MFLUCC 17-2658	99.82	*Diaporthe*
	1-5-25	PX856404	*Cercospora musigena* CPC 24809	100	*Cercospora*
	1-5-28	PX856405	*Cercospora glycinicola* CPC 23912	100	*Cercospora*
	1-5-3	PX856406	*Diaporthe rosae* MFLUCC 17-2658	99.82	*Diaporthe*
	1-5-4	PX856396	*Pseudocercospora pseudocydoniae* CBS:149392	100	*Pseudocercospora*
	1-5-5	PX856397	*Cercospora manoa* ICMP 21762	100	*Cercospora*
	1-5-6	PX856434	*Nemania aquilariae* KUMCC 20-0268	100	*Nemania*
	1-5-9	PX856398	*Diaporthe miriciae* BRIP 54736j	99.65	*Diaporthe*
	1-6-1	PX856399	*Cercospora musigena* CPC 24809	100	*Cercospora*
	1-6-1-1	PX856407	*Colletotrichum rhizophorae* MFLUCC 17-1927	100	*Colletotrichum*
	1-6-1-2	PX856408	*Cercospora manoa* ICMP 21762	100	*Cercospora*
	1-5-2-1	PX856409	*Cercospora manoa* ICMP 21762	100	*Cercospora*
	1-6-4	PX856410	*Periconia celtidis* MFLU 19-2784	100	*Periconia*
	1-6-5	PX856411	*Cercospora manoa* ICMP 21762	100	*Cercospora*
	1-7	PX856412	*Pseudopithomyces palmicola* MFLUCC 14-0392	100	*Pseudopithomyces*
	1-7-1	PX856413	*Cercospora manoa* ICMP 21762	100	*Cercospora*
	1-7-10	PX856417	*Cercospora musigena* CPC 24809	100	*Cercospora*
	1-7-10-1	PX856418	*Cercospora musigena* CPC 24809	100	*Cercospora*
	1-7-2	PX856414	*Cercospora manoa* ICMP 21762	100	*Cercospora*
	1-7-8	PX856415	*Nodulisporium verrucosum* CBS:245.29	99.80	*Nodulisporium*
	1-7-9	PX856416	*Cercospora manoa* ICMP 21762	100	*Cercospora*
	1-8	PX856419	*Colletotrichum siamense* ICMP:18578	100	*Colletotrichum*
	1-8-1	PX856420	*Colletotrichum siamense* ICMP:18578	100	*Colletotrichum*
Site 2	2-1	PX856435	*Xylaria feejeensis* AMIWEF-26	100	*Xylaria*
	2-10	PX856471	*Xylaria grammica* D15b3a	99.83	*Xylaria*
	2-11	PX856472	*Hypoxylon ferrugineum* SUT070	98.65	*Hypoxylon*
	2-1-11	PX856439	*Pseudocercospora nephrolepidicola* CBS 128211	99.79	*Pseudocercospora*
	2-1-12	PX856440	*Corynespora cassiicola* LPS-58	100	*Corynespora*
	2-1-14-1	PX856441	*Cercospora manoa* ICMP 21762	100	*Cercospora*
	2-1-15	PX856442	*Colletotrichum chiangmaiense* MFLUCC 18-0945	100	*Colletotrichum*
	2-1-18	PX856443	*Corynespora cassiicola* KACC 411262	100	*Corynespora*
	2-12	PX856473	*Colletotrichum rhizophorae* MFLUCC 17-1927	100	*Colletotrichum*
	2-1-24	PX856444	*Corynespora cassiicola* LPS-83	100	*Corynespora*
	2-1-3	PX856436	*Hypoxylon cocois* MFLU 23-0249	99.79	*Hypoxylon*
	2-13-1	PX856474	*Daldinia ehretiae* SAUCC228302	99.46	*Daldinia*
	2-1-31	PX856445	*Daldinia jianfengensis* SAUCC3738-4	98.77	*Daldinia*
	2-14	PX856475	*Nodulisporium verrucosum* CBS:245.29	99.80	*Nodulisporium*
	2-1-4-1	PX856437	*Colletotrichum plurivorum* CBS 12547	99.81	*Colletotrichum*
	2-16	PX856476	*Hypoxylon blackburniae* BRIP 72467b	98.86	*Hypoxylon*
	2-19	PX856477	*Hypoxylon cocois* MFLU 23-0249	99.80	*Hypoxylon*
	2-1-9	PX856438	*Cercospora musigena* CPC 24809	100	*Cercospora*
	2-2	PX856446	*Apiospora chromolaenae* MFLUCC 17-1505	99.66	*Apiospora*
	2-20	PX856478	*Cercospora musigena* CPC 24809	100	*Cercospora*
	2-22	PX856479	*Hypoxylon blackburniae* KUNCC24-18825	99.56	*Hypoxylon*
	2-2-20	PX856449	*Colletotrichum demersi* YMF 1.04946	100	*Colletotrichum*
	2-2-2-1	PX856447	*Daldinia jianfengensis* SAUCC373804	99.10	*Daldinia*
	2-2-23	PX856450	*Xylaria berteroi* 95101511	100	*Xylaria*
	2-23	PX856480	*Hypoxylon cocois* MFLU 23-0249	100	*Hypoxylon*
	2-25	PX856481	*Hypoxylon blackburniae* BRIP 72467b	98.30	*Hypoxylon*
	2-2-5	PX856448	*Hypoxylon cocois* MFLU 23-0249	100	*Hypoxylon*
	2-27	PX856482	*Hypoxylon blackburniae* BRIP 72467b	97.90	*Hypoxylon*
	2-28	PX856483	*Hypoxylon fendleri* BCC32408	99.31	*Hypoxylon*
	2-3	PX856451	*Pseudocercospora nephrolepidicola* CBS:128211	99.80	*Pseudocercospora*
	2-30	PX856484	*Corynespora cassiicola* Y01	100	*Corynespora*
	2-31	PX856485	*Colletotrichum demersi* YMF 1.04946	100	*Colletotrichum*
	2-3-14	PX856455	*Pseudopithomyces palmicola* MFLUCC 14-0392	100	*Pseudopithomyces*
	2-3-16	PX856456	*Hypoxylon cocois* MFLU 23-0249	99.80	*Hypoxylon*
	2-3-18	PX856457	*Paecilomyces lilacinus* NRRL895	100	*Paecilomyces*
	2-3-19	PX856458	*Corynespora cassiicola* LPS-58	100	*Corynespora*
	2-3-20	PX856459	*Cercospora manoa* ICMP 21762	100	*Cercospora*
	2-3-22	PX856460	*Colletotrichum plurivorum* CBS 125474	99.81	*Colletotrichum*
	2-3-2-2	PX856452	*Alternaria limicola* CBS 483.90	99.81	*Alternaria*
	2-3-24	PX856461	*Daldinia chiangdaoensis* BCC88221	100	*Daldinia*
	2-3-4	PX856453	*Cercospora musigena* CPC 24809	100	*Cercospora*
	2-3-7-1	PX856454	*Daldinia ehretiae* SAUCC228302	99.11	*Daldinia*
	2-4-2	PX856462	*Colletotrichum plurivorum* CBS 125474	99.81	*Colletotrichum*
	2-4-3	PX856463	*Colletotrichum guajavae* IMI 350839	100	*Colletotrichum*
	2-4-6	PX856464	*Colletotrichum plurivorum* CBS 125474	100	*Colletotrichum*
	2-4-8	PX856465	*Colletotrichum pandanicola* MFLUCC 17-0571	100	*Colletotrichum*
	2-5	PX856466	*Pestalotiopsis oryzae* CBS 353.69	100	*Pestalotiopsis*
	2-5-1	PX856467	*Diaporthe phaseolorum* M009B	85.08	Unrecognized genus
	2-7	PX856468	*Coprinellus micaceus* aislar 10.1a(2)_F	99.32	*Coprinellus*
	2-8	PX856469	*Colletotrichum cymbidiicola* IMI 347923	100	*Colletotrichum*
	2-8-2-1	PX856470	*Acrocalymma chuxiongense* AES-E01299B	100	*Acrocalymma*
Site 3	IN10-4-1	PX856505	*Alternaria angustiovoidea* CBS:195.86	100	*Alternaria*
	IN10-4-2	PX856506	*Nigrospora musae* CBS:319.34	99.82	*Nigrospora*
	IN10-5-3	PX856507	*Alternaria angustiovoidea* CBS:195.86	100	*Alternaria*
	IN1-2-2	PX856486	*Nigrospora hainanensis* CGMCC 3.18129	99.81	*Nigrospora*
	IN13	PX856508	*Cladosporium tenuissimum* CBS:125995	100	*Cladosporium*
	IN1-4-4	PX856487	*Neopestalotiopsis nebuloides* BRIP 66617	100	*Neopestalotiopsis*
	IN20	PX856509	*Colletotrichum demersi* YMF 1.04946	99.66	*Colletotrichum*
	IN22	PX856510	*Nigrospora saccharicola* CGMCC3.19362	99.69	*Nigrospora*
	IN2-2-1-1	PX856488	*Periconia imperatae* CGMCC 3.23931	99.65	*Periconia*
	IN24	PX856511	*Colletotrichum demersi* YMF 1.04946	100	*Colletotrichum*
	IN2-4-3-1	PX856489	*Alternaria angustiovoidea* CBS:195.86	100	*Alternaria*
	IN26	PX856512	*Phyllosticta capitalensis* CBS 128856	100	*Phyllosticta*
	IN27	PX856513	*Alternaria pipionipisi* CBS 116115	100	*Alternaria*
	IN31	PX856514	*Pseudopithomyces palmicola* MFLUCC 14-0392	100	*Pseudopithomyces*
	IN32	PX856515	*Alternaria angustiovoidea* CBS:195.86	99.64	*Alternaria*
	IN3-2-2	PX856490	*Flavodon flavus* IAvH-TRI-MR005	100	*Flavodon*
	IN35	PX856516	*Alternaria angustiovoidea* CBS:195.86	100	*Alternaria*
	IN37	PX856517	*Fusarium falciforme* CBS:475.67	100	*Fusarium*
	IN38	PX856518	*Pseudopithomyces maydicus* ICMP 12893	100	*Pseudopithomyces*
	IN41	PX856519	*Nigrospora osmanthi* CGMCC 3.18126	100	*Nigrospora*
	IN42	PX856520	*Paecilomyces lilacinus* NRRL895	100	*Paecilomyces*
	IN46	PX856521	*Didymella oligotrophica* CGMCC3.18111	100	*Didymella*
	IN48	PX856522	*Alternaria angustiovoidea* CBS:195.86	100	*Alternaria*
	IN5	PX856491	*Pseudopestalotiopsis avicenniae* MFLUCC 17-0434	100	*Pseudopestalotiopsis*
	IN52	PX856523	*Rhinocladiella similis* CBS 111763	100	*Rhinocladiella*
	IN5-2-1	PX856492	*Nigrospora musae* CBS:319.34	99.45	*Nigrospora*
	IN54	PX856524	*Nigrospora tomentosae* ZHKUCC 22-0339	100	*Nigrospora*
	IN56	PX856525	*Colletotrichum aeschynomenes* ICMP 17673	100	*Colletotrichum*
	IN58	PX856526	*Curvularia fraserae* BRIP 64708a	100	*Curvularia*
	IN6	PX856493	*Nigrospora osmanthi* CGMCC 3.18126	100	*Nigrospora*
	IN62-1	PX856527	*Nigrospora sphaerica* NS-6	100	*Nigrospora*
	IN63	PX856528	*Clonostachys krabiensis* MFLUCC 16-0254	99.40	*Clonostachys*
	IN64	PX856529	*Thyridium cornearis* CBS 131711	100	*Thyridium*
	IN6-5-3	PX856494	*Botrytis eucalypti* CERC 7170	100	*Botrytis*
	IN66	PX856530	*Clonostachys krabiensis* MFLUCC 16-0254	99.40	*Clonostachys*
	IN69	PX856531	*Nigrospora sphaerica* CN136E1	100	*Nigrospora*
	IN7-2-1	PX856495	*Periconia celtidis* MFLU 19-2784	99.06	*Periconia*
	IN7-2-3	PX856496	*Purpureocillium lilacinum* NRRL 895	100	*Purpureocillium*
	IN7-2-3-1	PX856497	*Alternaria angustiovoidea* CBS:195.86	100	*Alternaria*
	IN7-3-1	PX856498	*Epicoccum dendrobii* CGMCC 3.18359	100	*Epicoccum*
	IN7-4-3	PX856499	*Cladosporium benschii* COAD 2263	100	*Cladosporium*
	IN8	PX856500	*Exserohilum mcginnisii* CBS:325.87	99.83	*Exserohilum*
	IN8-5-3	PX856501	*Alternaria pogostemonis* ZHKUCC22-0146	100	*Alternaria*
	IN9-1-1	PX856502	*Nigrospora sphaerica* CN136E1	100	*Nigrospora*
	IN9-2-1	PX856503	*Alternaria angustiovoidea* CBS:195.86	100	*Alternaria*
	IN9-2-4	PX856504	*Nigrospora macarangae* MFLU 18-2518	100	*Nigrospora*

**Figure 4:**
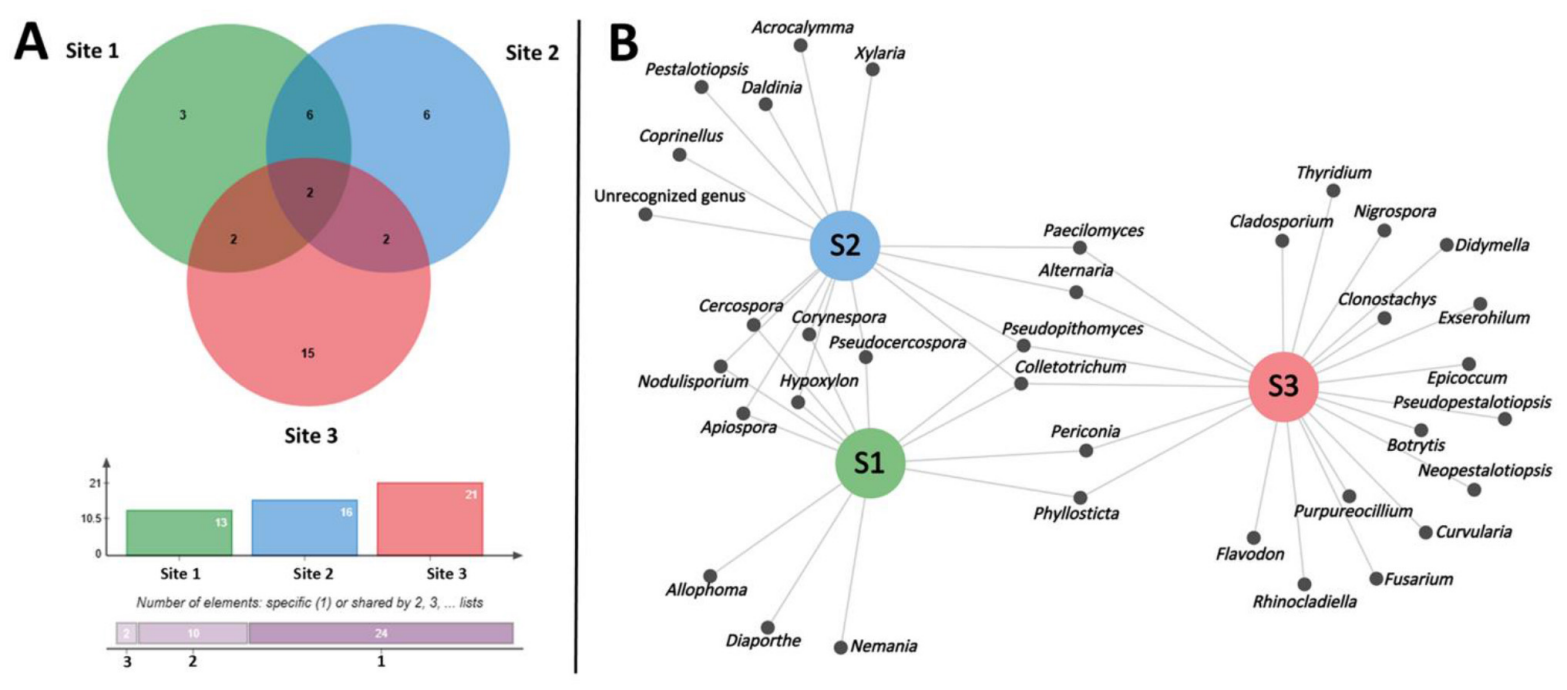
Number of fungal genera isolated from the different sampling sites in this study and their similarities **(A)** and network visualization of generic composition **(B)**. Site 1: Chiang Dao District, Chiang Mai Province, Thailand; Site 2: Fang District, Chiang Mai Province, Thailand; Site 3: Honghe Prefecture, Yunnan Province, China.

Comparison among the different sites indicated that the highest number of fungal genera was found at site 3 (21 genera), followed by site 2 (16 genera) and site 1 (13 genera; Figure 3B). *Cercospora* was the dominant genus at site 1, accounting for 39.4% of the isolates, followed by *Diaporthe* (18.3%) and *Colletotrichum* (15.5%). At site 2, *Hypoxylon* was the most abundant genus, comprising 23.5%, followed by *Colletotrichum* (21.6%), *Cercospora* (9.8%), and *Corynespora* (9.8%). At site 3, *Nigrospora* was the dominant genus, accounting for 23.9% of the isolates, followed by *Alternaria* (21.7%) and *Colletotrichum* (6.5%). The Venn diagram illustrating the overlap and unique fungal genera isolated from the different sampling sites is shown in Figure 4. The genera shared across all three sites were *Colletotrichum* and *Pseudopithomyces*. Six genera, including *Apiospora, Cercospora, Corynespora, Nodulisporium, Hypoxylon*, and *Pseudocercospora* were similar between site 1 and site 2. *Periconia* and *Phyllosticta* were similarly found in both site 1 and site 3. Notably, two genera, *Alternaria* and *Paecilomyces*, were found in both site 2 and site 3. The results indicated that the similarity of fungal genera between the two sites in Thailand (site 1 and site 2) was higher than the similarity between Thailand and China (site 1 and site 3; site 2 and site 3). Additionally, site 3 (Yunnan, China) had a higher number of unique fungal genera compared to site 1 and site 2 (Thailand). This suggests compositional differences in the endophytic fungal assemblages associated with Inca nut plants across the geographical sites examined in this study.

### 3.2 Determination of plant growth promoting properties

#### 3.2.1 Siderophore production

**Figure 5:**
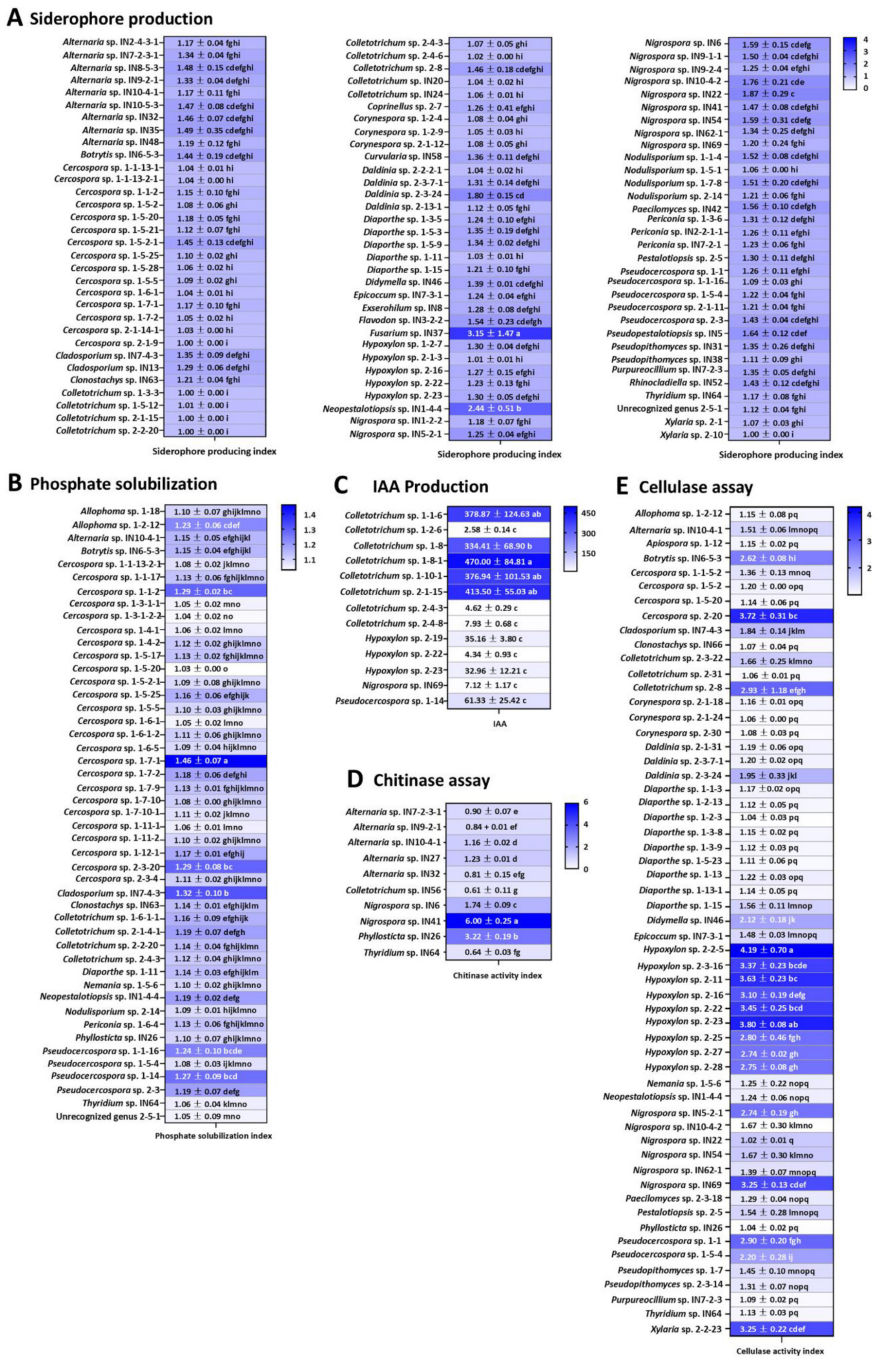
Heat maps of plant growth promoting properties of endophytic fungi obtained from Inca nut plants in this study: siderophore production **(A)**, phosphate solubilization **(B)**, IAA production **(C)**, chitinase activity **(D)**, and cellulase activity **(E)**.

Of the 168 fungal strains examined, 96 strains (57.1%) exhibited siderophore production, as indicated by the formation of yellow, orange, or pink color-change zones around the colonies on CAS agar. These positive productions were distributed among 30 distinct genera *viz. Alternaria, Botrytis, Cercospora, Cladosporium, Clonostachys, Colletotrichum, Coprinellus, Curvularia, Daldinia, Diaporthe, Epicoccum, Exserohilum, Flavodon, Fusarium, Hypoxylon, Neopestalotiopsis, Nigrospora, Nodulisporium, Paecilomyces, Paraboeremia, Periconia, Pestalotiopsis, Pseudocercospora, Pseudopestalotiopsis, Pseudopithomyces, Purpureocillium, Pyricularia, Rhinocladiella, Thyridium*, and *Xylaria*; as well as one unrecognized genus represented by strain 2-5-1. Quantitative evaluation based on the siderophore production index (SPI) revealed substantial variation in siderophore-producing capacity among the fungal strains (Figure 5A). The SPI values ranged from 1.00 to 3.15. *Fusarium* sp. IN37 exhibited the highest SPI value, indicating the strongest siderophore producing potential among all positive strains, followed by *Neopestalotiopsis* sp. IN1-4-4, *Nigrospora* sp. IN22. and *Daldinia* sp. 2-3-24.

#### 3.2.2 Phosphate solubilization

The capability of endophytic fungal strains to solubilize insoluble phosphate was assessed by observing the formation of clear halos around fungal colonies on PVK agar. Among the 168 tested strains, 47 strains (28.0%) demonstrated phosphate-solubilizing activity, as evidenced by the production of distinct clear zones surrounding the colonies. These positive isolates were distributed across 15 genera, including *Allophoma, Alternaria, Botrytis, Cercospora, Cladosporium, Clonostachys, Colletotrichum, Diaporthe, Nemania, Neopestalotiopsis, Nodulisporium, Periconia, Phyllosticta, Pseudocercospora*, and *Thyridium*, as well as strain 2-5-1. The results indicated that phosphate solubilization ability varied considerably among fungal strains, as evidenced by differences in the phosphate solubilization index (PSI; Figure 5B). PSI values ranged from 1.03 to 1.46. *Cercospora* sp. 1-7-1 shows the strongest ability to solubilize the insoluble phosphate, followed by *Cladosporium* sp. IN7-4-3 and *Cercospora* sp. 2-3-20.

#### 3.2.3 Indole compound production and IAA quantification

**Figure 6:**
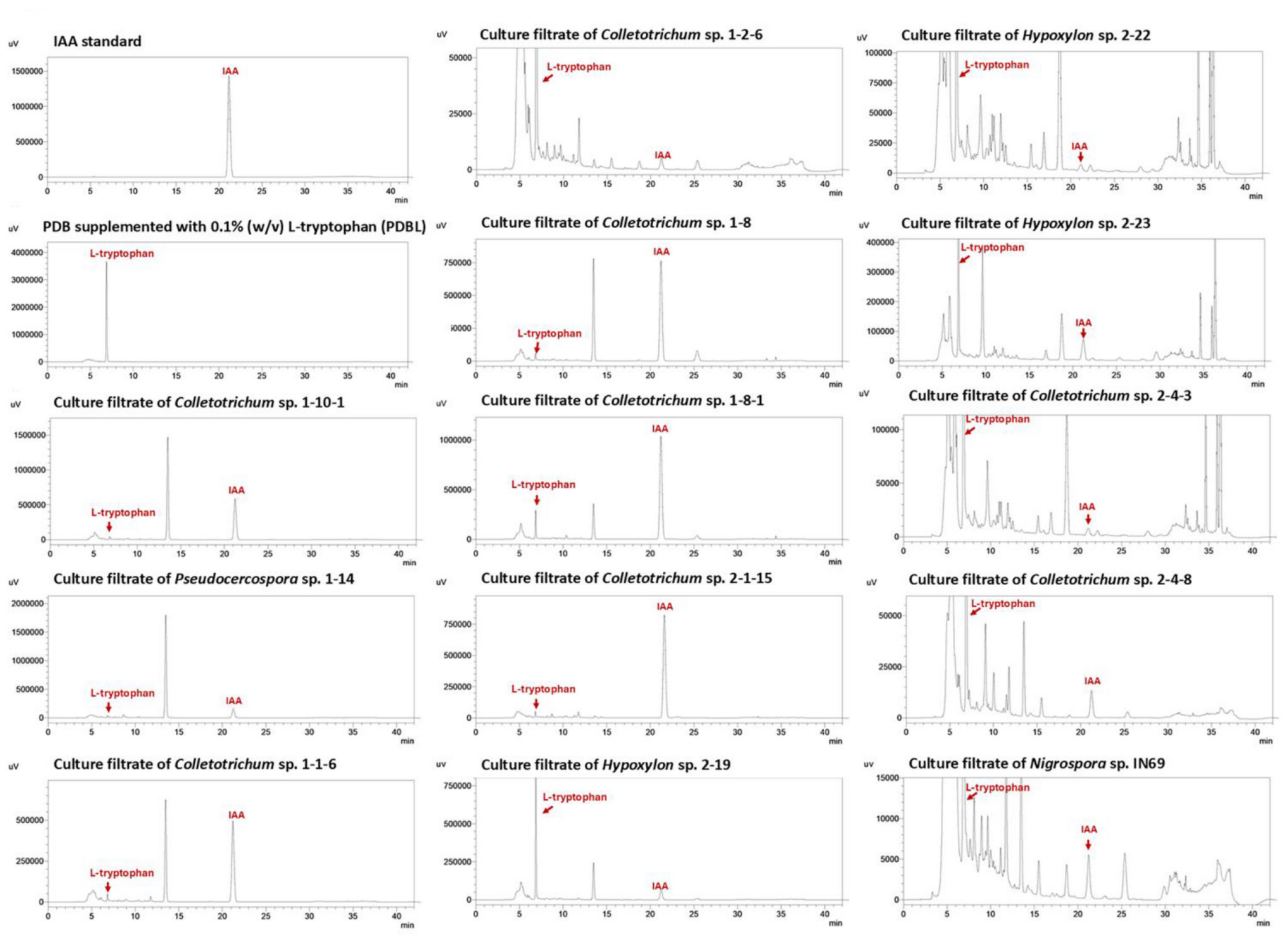
High-performance liquid chromatogram showing identification of indole-3-acetic acid in fungal liquid cultures of endophytic fungi isolated from Inca nut plants with positive indole compound production.

Of the 168 fungal strains examined, 13 strains (7.7%) displayed positive indole compound production by chromogenic reaction to Salkowski's reagent, as indicated by the formation of a distinct pink-to-red color. These 13 strains belonged to the genera *Colletotrichum* (eight strains), *Hypoxylon* (three strains), *Nigrospora* (one strain), and *Pseudocercospora* (one strain). Subsequently, IAA production was confirmed and quantified by HPLC analysis using authentic IAA standard. HPLC analysis confirmed IAA production by the fungal strains, as evidenced by peaks matching the authentic IAA standard at a retention time of 21.1 min (Figure 6) and maximum absorbance at 279 nm, consistent with (Kumla et al. 2020). IAA production from the 13 positive fungal strains ranged from 2.58 to 470.00 μg mL^−1^, which varied depending on the fungal strain (Figure 5C). *Colletotrichum* sp. 1-8-1 produced the highest IAA level, while *Colletotrichum* sp. 1-2-6 showed the lowest level.

### 3.3 Extracellular enzyme production

The ability of all 168 fungal strains to produce extracellular enzymes, including cellulase and chitinase was determined by agar plate assay and expressed as an enzyme activity index (EAI). The EAI values revealed variation in enzyme-producing capacity among the fungal strains (Figures 5D, E). The results indicated that 57 (33.9%) strains, distributed across 25 genera (*Allophoma, Alternaria, Apiospora, Botrytis, Cercospora, Cladosporium, Clonostachys, Colletotrichum, Daldinia, Diaporthe, Epicoccum, Hypoxylon, Nemania, Neopestalotiopsis, Nigrospora, Paecilomyces, Paraboeremia, Pestalotiopsis, Phyllosticta, Pseudocercospora, Pseudopithomyces, Purpureocillium, Pyricularia, Thyridium*, and *Xylaria*), could produce cellulase, while 10 (6.0%) strains from five genera (*Alternaria, Colletotrichum, Nigrospora, Phyllosticta*, and *Thyridium*) could produce chitinase. The highest EAI values for cellulase and chitinase were obtained from *Hypoxylon* sp. 2-2-5 and *Nigrospora* sp. IN41, respectively.

### 3.4 Plant growth promoting effects of selected fungal culture filtrates containing IAA

#### 3.4.1 Seed germination

**Figure 7:**
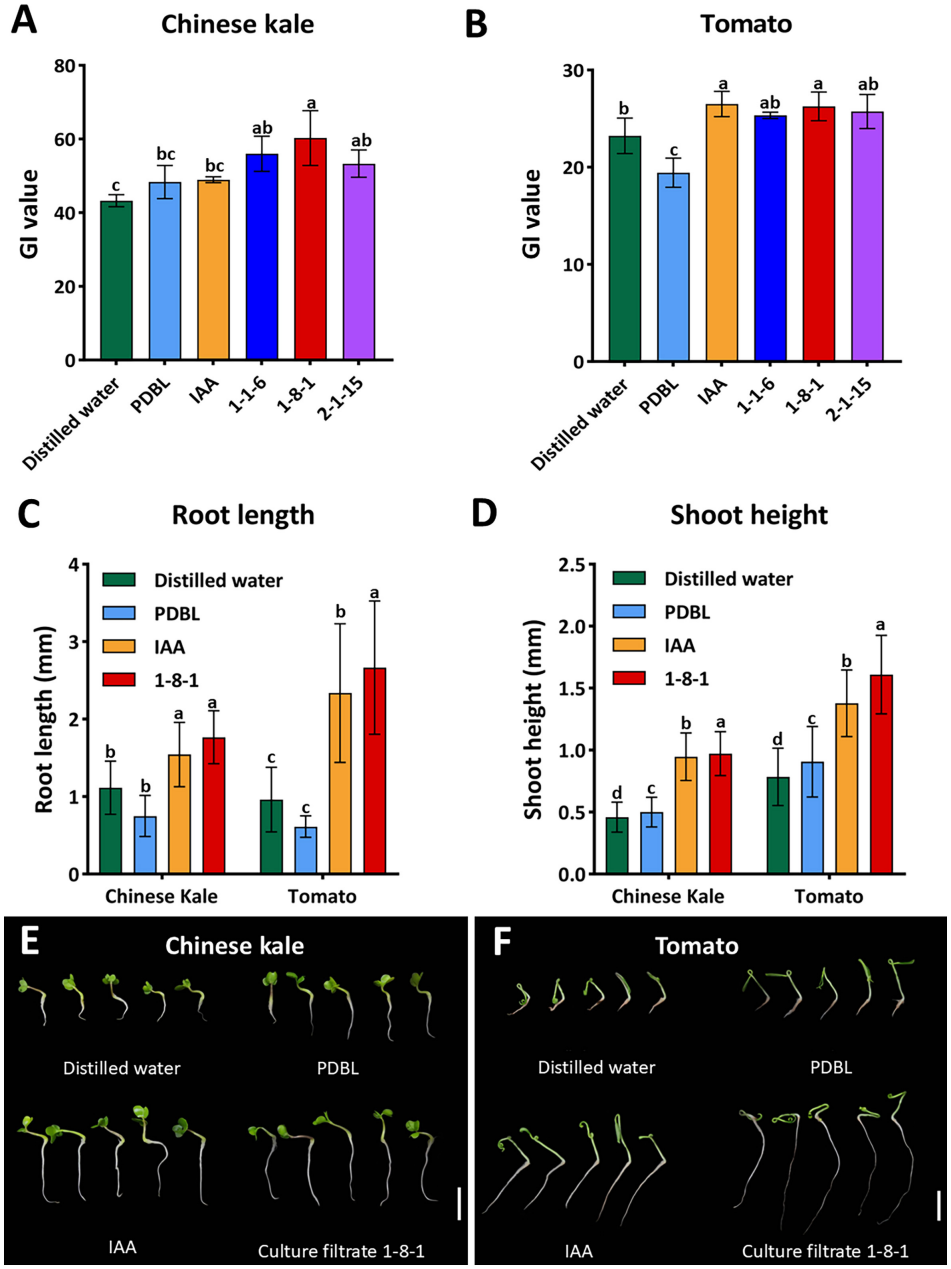
Seed germination index **(A, B)** and seedling growth **(C–F)** of Chinese kale and tomato after treatment with distilled water, PDB supplemented with 0.1% L-tryptophan (PDBL), 25 μg mL^−1^ IAA, and selected fungal culture filtrates containing 25 μg mL^−1^ IAA from *Colletotrichum* strains 1-1-6, 1-8-1, and 2-1-15. Scale bars in E and F = 10 mm.

Three culture filtrates of *Colletotrichum* strains 1-1-6, 1-8-1, and 2-1-15 were used to assess the effect of fungal IAA on seed germination. The positive control consisted of authentic IAA, while the negative controls included sterile distilled water and PDB supplemented with 0.1% (*w*/*v*) L-tryptophan (PDBL). The germination index (GI) for each plant was calculated and it is presented in Figures 7A, B. Chinese kale seeds treated with sterile distilled water and PDBL showed relatively low GI values (43.26 ± 1.63 and 48.34 ± 4.49, respectively), similar to authentic IAA treatment (48.97 ± 2.77). However, all fungal culture filtrates significantly increased the GI values of Chinese kale seeds (53.31 ± 3.71 to 60.29 ± 7.42). The highest GI value was observed in culture filtrates of strain 1-8-1. In tomato, the GI values of seeds treated with authentic IAA (26.52 ± 1.31) and fungal culture filtrates from three strains (25.34 ± 1.75 to 26.26 ± 1.46) did not differ significantly among these treatments. However, all were significantly higher than those of the sterile distilled water (23.24 ± 1.83) and PDBL (19.44 ± 1.50) controls. Therefore, fungal culture filtrates containing IAA from *Colletotrichum* sp. 1-1-6, 1-8-1, and 2-1-15 effectively enhanced seed germination in both Chinese kale and tomato.

#### 3.4.2 Seedling growth

The culture filtrate of *Colletotrichum* strain 1-8-1 was used to assess the effect of fungal IAA on seedling growth, as exhibited the highest seed germination index. Regarding seedling growth, fungal culture filtrate from strain 1-8-1 significantly increased the root length of Chinese kale and tomato, with no significant difference compared to authentic IAA (Figures 7C, E, F). However, both treatments showed significantly longer roots than the sterile distilled water and PDBL controls. Both Chinese kale and tomato seedlings showed the shortest roots in the distilled water control. Additionally, both the fungal culture filtrate and authentic IAA significantly increased shoot height in Chinese kale, with no significant difference between these treatments; however, both were significantly higher than the distilled water and PDBL controls (Figures 7D, E). In tomato seedlings, those treated with fungal culture filtrate exhibited the greatest shoot height, followed in decreasing order by authentic IAA, the PDBL control, and the sterile distilled water control (Figures 7D, F).

## 4 Discussion

Research on endophytic fungi has attracted growing interest owing to their ecological significance in plant health and their potential agricultural applications (Baron and Rigobelo, 2022; Grabka et al., 2022; Tariq et al., 2025). In this study, we preliminarily identified the endophytic fungi associated with healthy leaves of Inca nut plants (*Plukenetia volubilis*) collected in Yunnan, China and northern Thailand. These results are consistent with previous studies, which report that endophytic Ascomycota occur more frequently than Basidiomycota due to several ecological and biological advantages (Rashmi et al., 2019; Rungjindamai and Jones, 2024). Ascomycota produce abundant, easily dispersed asexual spores, enabling them to colonize plant tissues more rapidly and frequently than Basidiomycota, and they are well adapted to asymptomatic growth within plant tissues (Morrow and Fraser, 2009; Rungjindamai and Jones, 2024). In contrast, most Basidiomycota are primarily saprotrophic or pathogenic and are less commonly found as endophytes (Schmidt-Dannert, 2016; Rungjindamai and Jones, 2024). In this study, most endophytic fungi were found to belong to the genera *Alternaria, Cercospora, Colletotrichum, Corynespora, Diaporthe, Hypoxylon*, and *Nigrospora*, consistent with previous studies, which have shown that these genera are abundant and frequently encountered as endophytes in various plant species in China and Thailand (Cui et al., 2022; Onlamun et al., 2023; Photita et al., 2001; Suwannarach et al., 2012; Yang et al., 2018; Zhao et al., 2020). As a whole, the endophytic fungal community composition of Inca nut analyzed in this study carried out in China and Thailand proved to be markedly different from those reported by (Márquez-Dávila et al. 2020) in Peru, who investigated the endophytic fungi from seven *Plukenetia* species, including Inca nut, and reported *Clonostachys, Xylaria, Pestalotiopsis*, and *Trichoderma* as the dominant genera. Our findings are consistent with numerous previous studies showing that differences in endophytic fungal community composition are attributable to geographic variation (Langenfeld et al., 2013; Martins et al., 2016; Wu et al., 2013). Further study is required for species identification of all endophytic fungal strains obtained in this study using a multi-gene approach. This molecular characterization would involve sequencing multiple genetic markers to achieve accurate taxonomic resolution at the species level. A limitation of the present study is that it examined only the effects of geographic factors on endophytic fungal community composition. Other important ecological and environmental variables, including climate conditions, soil properties, and local biotic interactions, were not incorporated into the analysis. Further research should consider other environmental and ecological variables to gain a more comprehensive understanding of the composition of endophytic fungal communities.

Endophytic fungi promote plant growth by enhancing nutrient acquisition through siderophore production and phosphate solubilization, producing phytohormones, and secreting cell wall-degrading enzymes, with these abilities varying among species and strains (Baron and Rigobelo, 2022; Tabbasum et al., 2022). Siderophore production is one of the key mechanisms by which plant-associated fungi promote plant growth by enhancing iron acquisition under iron-limited conditions and by suppressing phytopathogens through competitive iron sequestration (Brick et al., 2025; Tabbasum et al., 2022). In this study, 96 endophytic fungal strains exhibited positive siderophore production on CAS agar. This outcome was consistent with prior studies that found that some species and strains of endophytic fungi (e.g., genera *Alternaria, Colletotrichum, Fusarium*, and *Nigrospora*) could produce siderophores (Al-Sissi et al., 2025; Ratul et al., 2023; Roy et al., 2021; Sodhi and Saxena, 2023b). In this study, *Fusarium* strain IN37 exhibited the most effective siderophore production among the tested strains. Similarly, siderophore production has been reported in endophytic *Fusarium* species isolated from tea (*Camellia sinensis*), creeping woodsorrel (*Oxalis corniculata*), and Arabian jasmine (*Jasminum sambac*; Al-Harthi et al., 2023; Ratul et al., 2023). Subsequently, 47 endophytic fungal strains isolated from Inca nut plants solubilized insoluble phosphate on agar plates with solubilization indices ranging from 1.03 to 1.46. These results support previous reports of phosphate-solubilizing capacity in endophytic fungi, with reported solubilization indices ranging from 1.00 to 4.29 depending on the fungal strains (da Silva Ribeiro et al., 2021; dos Santos Oliveira et al., 2021; Rosli et al., 2020; Sahoo and Gupta, 2018). In this study, endophytic fungal strains belonging to the genera *Cercospora, Cladosporium*, and *Pseudocercospora* demonstrated the most effective solubilization of insoluble phosphate among all tested strains. This finding is consistent with previous reports of phosphate-solubilizing capacity in *Cladosporium* (Sahoo and Gupta, 2018; Sawant and Rodrigues, 2020). However, this is the first report documenting phosphate-solubilizing capacity in *Cercospora* and *Pseudocercospora*. In this study, endophytic fungi isolated from Inca nut plants demonstrated the ability to produce siderophores and solubilize phosphate; however, further studies are needed to characterize the specific types of siderophores (e.g., hydroxamate-, carboxylate-, or catecholate-type) and to elucidate the underlying mechanisms of phosphate solubilization, including organic acid secretion, chelation, and hydrolytic enzyme activity.

Endophytic fungi that produce extracellular cellulolytic and chitinolytic enzymes provide multiple benefits to their host plants. Chitinases degrade chitin, a major structural component of fungal cell walls, thereby inhibiting the growth of phytopathogenic fungi and contributing to biological disease control, while cellulases weaken pathogen cell walls and disrupt microbial structures by enzymatically breaking down cellulose present in the cell walls of oomycetes and some bacterial pathogens (Malik et al., 2023; Martínez-Zavala et al., 2020). This study demonstrated that 57 strains (belonging to genera *Allophoma, Alternaria, Apiospora, Botrytis, Cercospora, Cladosporium, Clonostachys, Colletotrichum, Daldinia, Diaporthe, Epicoccum, Hypoxylon, Nemania, Neopestalotiopsis, Nigrospora, Paecilomyces, Paraboeremia, Parahypoxylon, Pestalotiopsis, Phyllosticta, Pseudocercospora, Pseudopithomyces, Purpureocillium, Pyricularia, Thyridium*, and *Xylaria*) and 10 strains (belonging to genera *Alternaria, Colletotrichum, Nigrospora, Phyllosticta*, and *Thyridium*) exhibited the ability to produce cellulase and chitinase, respectively, as determined by the agar plate assays. These findings are consistent with previous studies reporting that endophytic fungi commonly secrete extracellular cellulases and chitinases, with enzyme production varying among fungal species and strains (Ali et al., 2019; Batista et al., 2022; Kumar and Prasher, 2022; Mwendwa et al., 2025). *Hypoxylon* sp. 2-2-5 and *Nigrospora* sp. IN41 demonstrated the highest cellulase and chitinase activities, respectively, consistent with previous findings documenting cellulase and chitinase production in these genera (Maxwell et al., 2018; Maheshwari and Mmbaga, 2024; Mtui, 2012). Generally, cellulases and chitinases produced by endophytic fungi are associated with antagonistic activity against phytopathogens. Therefore, further studies should investigate their inhibitory effects against a range of plant pathogens under laboratory and greenhouse conditions to evaluate their potential application as biological control agents.

IAA production occurs not only in plants but also in certain microorganisms, with L-tryptophan serving as the primary precursor in most microbial IAA production (Duca et al., 2014; Numponsak et al., 2018; Tang J. et al., 2023). This study revealed that 13 fungal strains across four genera, *Colletotrichum, Hypoxylon, Nigrospora*, and *Pseudocercospora*, synthesized IAA, with concentrations ranging from 2.58 to 470.00 μg mL^−1^. These findings are consistent with numerous previous studies reporting that endophytic fungi can produce IAA in the presence of L-tryptophan, with IAA levels varying depending on fungal species and strains (Turbat et al., 2020; Sodhi and Saxena, 2023a; Taheri, 2025). The IAA levels obtained in this study fall within the range reported in previous studies, from 0.30 to 662.96 μg mL^−1^ (Jagannath et al., 2019; Numponsak et al., 2018; Sodhi and Saxena, 2023a; Wang et al., 2024). Strains belonging to the genus *Colletotrichum* exhibited the highest IAA production, consistent with previous reports documenting IAA production by endophytic *Colletotrichum* (Dutta and Banerjee, 2024; Mukherjee et al., 2022; Numponsak et al., 2018; Wary et al., 2022). In this study, the culture supernatant containing IAA produced by *Colletotrichum* sp. 1-8-1 significantly enhanced seed germination, root length, and shoot height in both Chinese kale and tomato. Similarly, the culture extract from endophytic fungus, *C. gloeosporioides* contain IAA significantly increased seed germination percentage of *Aerides houlletiana* compared with control treatments (Suwattanachat et al., 2022). Previous studies have also reported that culture filtrates and culture extracts containing IAA from endophytic fungi significantly enhanced germination and growth parameters in plant seedlings (Abdelhamid et al., 2024; García-Latorre et al., 2023; Turbat et al., 2020). Further studies are required to identify the IAA biosynthetic pathways and optimize culture conditions for enhanced IAA production, as well as to evaluate the potential application of *Colletotrichum* sp. 1-8-1 and its culture filtrate as plant growth-promoting agents across diverse plant species, including Inca nut, under both greenhouse and field conditions.

## 5 Conclusion

This study is the first investigation of endophytic fungi associated with Inca nut plants (*P*. *volubilis*) in China and Thailand. One hundred sixty-eight fungal strains were obtained and identified based on the ITS marker gene, belonging to 35 previously known genera and one unrecognized genus. Among these, Ascomycota predominated, accounting for 166 strains (98.8%), while two strains (1.2%) were Basidiomycota. Generic diversity was highest at the Honghe site (site 3), China. *Nigrospora* and *Cercospora* predominated at Honghe (site 3) and Chiang Dao (site 1), Thailand, respectively, while *Hypoxylon* dominated at Fang (site 2), Thailand. Notably, *Colletotrichum* and *Pseudopithomyces* were found at all sampling sites. Moreover, the obtained endophytic fungi demonstrated diverse plant growth promoting activities, including siderophore production (96 strains), phosphate solubilization (47 strains), IAA synthesis (13 strains), cellulase production (57 strains), and chitinase production (10 strains). Furthermore, fungal culture filtrates containing IAA from *Colletotrichum* strains 1-1-6, 1-8-1, and 2-1-15 enhanced seed germination in Chinese kale and tomato, with the culture filtrate from strain 1-8-1 also significantly promoting root and shoot growth in seedlings of both species. This study highlights the important ecological roles of these endophytes and their potential utility in sustainable agricultural applications. Further studies should focus on elucidating the molecular and biochemical mechanisms underlying these beneficial traits. Evaluation of plant growth promoting efficacy using fungal culture filtrates or fungal inocula under greenhouse and field conditions is also required, along with assessments of host specificity, ecological safety, and long-term effects on plant health. These steps are essential for developing endophytic fungi associated with Inca nut plants as effective and environmentally friendly biofertilizers or biostimulants.

## Data Availability

The datasets presented in this study can be found in online repositories. The names of the repository/repositories and accession number(s) can be found in the article.
